# Identification of Long Non-Coding RNA as Potential Biomarkers for the Diagnosis of Postmenopausal Osteoporosis

**DOI:** 10.1155/2024/8820697

**Published:** 2024-11-23

**Authors:** Zhu Yihua, Peng Chenjian, Wang Lining, Jiang Shujun, Yang Haomiao, Zhang Kaixuan, Dai Fenglei, Ma Yong, Chu Xudong, Zhang Chunlei, Sun Haitao

**Affiliations:** ^1^Laboratory of New Techniques of Restoration & Reconstruction, Institute of Traumatology & Orthopedics, Nanjing University of Chinese Medicine, Nanjing, China; ^2^Department of Orthopedic Surgery, Nanjing Hospital of Chinese Medicine Affiliated to Nanjing University of Chinese Medicine, Nanjing, China; ^3^School of Integrated Chinese and Western Medicine, Nanjing University of Chinese Medicine, Nanjing, China; ^4^Department of Infectious Diseases, Nanjing Hospital of Chinese Medicine Affiliated to Nanjing University of Chinese Medicine, Nanjing, China; ^5^Wuxi Affiliated Hospital of Nanjing University of Chinese Medicine, Jiangsu Province Traditional Chinese Medicine Retrogressive Osteoarthritis Clinical Medical Innovation Center, Wuxi, China; ^6^Department of Orthopedic Surgery, Affiliated Huishan Hospital of Xinglin College, Nantong University, Wuxi Huishan District People's Hospital, Wuxi 214100, China

**Keywords:** biomarker, lncRNA, postmenopausal osteoporosis, RNA sequencing

## Abstract

**Objective:** To explore the feasibility and clinical application value of differentially expressed lncRNA in human peripheral blood mononuclear cell (PBMC) as a potential biomarker for postmenopausal osteoporosis (PMOP).

**Methods:** In this study, a case-control trial was conducted to collect a total of 10 samples of PBMC from PMOP and postmenopausal-without-osteoporosis (n-PMOP) patients. RNA sequencing was performed to profile lncRNA and mRNA expression, identifying differentially expressed lncRNAs (DElncRNAs) and mRNAs (DEmRNAs) based on the criteria of fold change (FC) ≥ 2 and *p* value < 0.05; GO and KEGG enrichment analyses were carried out for differentially expressed genes (DEGs); 10 DElncRNAs and 20 DEmRNAs were selected for lncRNA–mRNA correlation analysis and Circos plot to screen out the lncRNAs that could be used as potential biomarkers. Then, ROC curve analysis was used to evaluate the diagnostic and therapeutic value of DElncRNAs as clinical potential diagnostic markers for PMOP. Afterward, 20 PMOP and 20 n-PMOP patients were reincluded and quantitative real-time PCR (qRT-PCR) was performed to externally validate the screening of lncRNAs.

**Result:** (1) Compared with n-PMOP, there were 1978 DElncRNAs and 1024 DEmRNAs in PMOP patients. (2) Bioinformatics technology was used to analyze the DEGs, and the GO analysis showed that the activities of the gene products were mainly related to the protein binding, membrane, plasma membrane, and extracellular region. The results of KEGG enrichment analysis showed that it was mainly enriched in PI3K-Akt signaling pathway, metabolic pathways, and pathways in cancer and focal adhesion. (3) The correlation network and Circos plot further indicated the implication of DElncRNA expression profiles in PMOP via interactions with DEmRNAs. Among them, lncRNA RAB37, lncRNA BEGAIN, and lncRNA ZNF529 had the highest number of nodes, totaling 19, possibly potential diagnostic markers for PMOP. (4) The diagnostic efficacy of the screened lncRNAs was analyzed by ROC curve. The results showed an the area under the ROC curve (AUC) of 0.960 for lncRNA RAB37, 1.000 for lncRNA ZNF529, 1.000 for lncRNA BEGAIN. (5) The qPCR results showed that lncRNA RAB37, lncRNA ZNF529, and lncRNA BEGAIN were all significantly correlated with the occurrence of PMOP (*p* < 0.05). However, the significant difference of lncRNA ZNF529 was superior to that of other lncRNAs.

**Conclusion:** The lncRNA ZNF529 is significantly overexpressed in PBMC in PMOP, and bioinformatics analysis and validation experiments indicate that it is closely associated with PMOP; thus, it is expected to be a potential diagnostic marker for PMOP.

## 1. Introduction

The aging of society will inevitably lead to an increase in degenerative diseases such as osteoporosis. The damage caused by osteoporosis is even more severe in postmenopausal women due to their lower peak bone mass and significant hormonal disturbances after menopause. About 20% of women over the age of 50 and 30% of women 65 or older in the United States meet the criteria for osteoporosis [1]. Postmenopausal osteoporosis (PMOP) is characterized by decreased bone mineral density (BMD) and degradation of bone microarchitecture, bone fragility, and increased fracture risk due to estrogen deficiency [2]. Over 2 million osteoporosis-related fractures occur in the United States each year, with associated costs predicted to be up to $25 billion by 2025 [3]. The high incidence of PMOP and its accompanying pain and risk of fracture are a serious threat to women's physical and mental health. Therefore, it is crucial to diagnose PMOP as early as possible to safeguard women's health, though this presents many challenges.

Delayed diagnosis of PMOP is common in clinical practice due to the lack of preliminary symptoms and typical features, resulting in lack of early intervention and early warning of fracture risk in patients with bone loss [4]. Although clinical examinations of bone turnover markers such as C-telopeptide of type I collagen (CTX-I) and N-terminal propeptide of type I procollagen (PINP) are common, with a positive role in evaluating the response to anabolic and anti-resorptive therapies, they are not useful for the diagnosis of osteoporosis, nor does it improve the prediction of an individual's fracture risk [5]. Currently, the clinical diagnosis of osteoporosis relies mainly on BMD as a diagnostic indicator. Although the BMD test has good diagnostic efficacy, it still has certain shortcomings. Firstly, osteoporosis is a complex pathological change associated with multiple factors such as genetics, aging, endocrinology, immunity, and intestinal flora, and clinical diagnosis should not be limited to BMD [6–8]. Secondly, positive imaging features tend to lag behind persistent abnormalities of bone metabolism, somewhat diminishing their value in early diagnosis [9]. In addition, dual energy X-ray absorptiometry tends to be expensive and not portable, requiring a dedicated site to house the equipment, less widely available [10]. Although the T-value has good diagnostic value, it is not a complete threshold for treatment decisions [11]. Therefore, in-depth exploration of potential diagnostic biomarkers of PMOP is important for osteoporosis diagnosis and treatment.

With the advancement of time and technological innovations, the emergence and maturity of high-throughput sequencing technology has given researchers powerful tools to translate genomic information and thus obtain valuable research targets [12]. In this context, the great heterogeneity of lncRNA-interacting molecules and patterns of expressed lncRNA revealed bring new perspectives for the diagnosis of PMOP [13]. lncRNAs are non-protein coding RNA transcripts over 200 nucleotides (200 bp∼100 kbp) [14]. lncRNAs affect multiple biological functions through interactions with RNA, DNA, and proteins such as RNA transcription and splicing, mRNA transport and translation, and protein post-translational modification [15]. The potential value of lncRNAs as novel biomarkers for early diagnosis, treatment, and prognostic monitoring has been well established in cancer [16], neurological [17], and bone metabolic diseases [18]. Recently, lncRNAs and osteoporosis have become increasingly relevant. Wu et al. [19] identified DOCK4 as a potential biomarker for elderly osteoporosis by integrative analysis of lncRNA and mRNA expression data. Yu et al. [20], in turn, found that the lncRNA MIAT could be used as a biomarker for fragility fractures and promoting fracture healing. lncRNAs may play a key role in the development and progression of osteoporosis by regulating gene expression, epigenetic modifications, cell signaling pathways, and other mechanisms [21]. The interaction of lncRNA HoxA-AS3 with Enhancer Of Zeste 2 is required for H3 lysine-27 trimethylation of the osteogenic transcription factor Runx2 [22]. lncRNA DANCR can interact directly with Brahma-related gene 1 to regulate Nfatc1 transcription and Pgc1 *β*-dependent metabolic shifts in osteoclastogenesis [23]. lncTIMP3 may promote bone marrow mesenchymal stem cell activity and enhance osteoblast differentiation via the miR-214/Smad4 axis, delaying the progression of PMOP [24]. Whether in disease diagnosis or treatment, lncRNAs show favorable applications and development prospects.

In this study, the objective was to identify specific lncRNAs associated with PMOP through RNA sequencing and evaluate their feasibility as potential biomarkers.

## 2. Materials and Methods

### 2.1. Participants

This study recruited 50 patients, 25 PMOP patients and 25 n-PMOP patients at Nanjing Hospital of C.M. from March 2023 to April 2024. The inclusion criteria were as follows: (i) aged between 55 and 85 years and menopausal for more than 1 year; (ii) diagnosis of osteoporosis based on the World Health Organization criteria [11]; (iii) having complete clinical data; and (iv) signed the informed consent form. The exclusion criteria were as follows: (i) any comorbidity that could significantly affect bone metabolism, such as endocrine disease, immune disease, and phosphorus metabolism disorder; (ii) previous participation in similar studies or treatment with anti-osteoporosis treatment; and (iii) serious heart, brain, liver, or kidney disease. In total, 50 unrelated women were eligible and included in the analysis. This study was approved by the Ethics Committee of Nanjing Hospital of C.M. (Approval number: KY2023254).

### 2.2. Study Design

**Figure 1:**
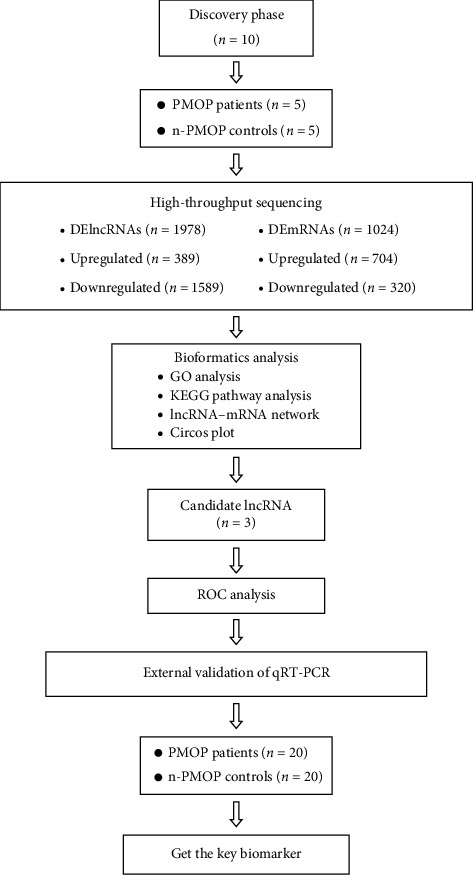
Flowchart of the study: Screen study participants and collect general clinical data. Afterward, peripheral blood mononuclear cell collection, RNA extraction, and library construction were performed. Screen potential lncRNAs using expression profiling data and bioinformatics analysis results. Subsequently, the diagnostic and therapeutic value of DElncRNAs was assessed by ROC curve analysis, and key lncRNAs were identified by external validation via qRT-PCR.

In this study, a case-control trial was conducted and participants were divided into 2 groups: the PMOP group and the n-PMOP group. The experiment was divided into two phases: discovery phase and validation phase (Figure 1).

### 2.3. Peripheral Blood Mononuclear Cell Acquisition (PBMC)

Blood samples were collected by venipuncture and mononuclear cells were extracted no later than 4 h after blood collection. Mononuclear cells were extracted from 2 mL whole blood using PBMC separation fluid (Tianjin Haoyang Biological Manufacture Co., Ltd., Tianjin, China) according to the manufacturer's protocol. Add TRIzol to lysed cells and store at −80°C for subsequent RNA extraction.

### 2.4. RNA Extraction and Library Construction

To extract and purify total RNA with TRIzol reagent (Invitrogen, Carlsbad, CA, USA). The quantity and purity of total RNA were analyzed by Bioanalyzer 2100 and RNA 6000 Nano LabChip Kit (Agilent, CA, USA, 5067–1511) with a RIN number greater than 7.0. Using the Epicentre Ribo-Zero Gold Kit (Illumina, cat. MRZG12345, San Diego, USA), ribosomal RNA was removed with approximately 5 *μ*g of total RNA. Subsequently, the ribo-minus RNA was fragmented into small pieces using Magnesium RNA Fragmentation Module (NEB, cat. e6150S, USA). Then, reversed transcription of the cleaved RNA fragments was performed with SuperScript™ II Reverse Transcriptase (Invitrogen, cat. 1896649, USA). U-labeled second-strand DNA was in turn synthesized by *E. coli* DNA polymerase I (NEB, cat. m0209, USA), RNase H (NEB, cat. m0297, USA), and dUTP solution (Thermo Fisher Scientific, cat. R0133, USA). Next, each strand has an A base added to the blunt ends of the strand. T-base overhangs are contained in each adapter and are used to ligate the adapter to the A-tailed fragment DNA. Attach single-indexed or dual-indexed adapters to the fragment and use AMPureXP beads for size selection (300∼600 bp). U-labeled second-stranded DNAs were treated first with heat-labile UDG enzyme (NEB, cat. m0280, USA), and the ligated product was amplified by PCR afterward (initial denaturation at 95°C for 3 min; 8 cycles of denaturation at 98°C for 15 s, annealing at 60°C for 15 s, and extension at 72°C for 30 s; and then final extension at 72°C for 5 min). The final cDNA library had an average insert size of 300 ± 50 bp. Finally, 2 × 150 bp paired-end sequencing (PE150) was performed on Illumina Novaseq™ 6000 (LC-Bio Technology CO., Ltd., Hangzhou, China) following the vendor's recommended protocol.

### 2.5. Bioinformatics Analysis

The reads were further filtered by Cutadapt (https://cutadapt.readthedocs.io/en/stable/,version:cutadapt-1.9) in order to get high-quality clean reads. Then sequence quality was verified using FastQC (http://www.bioinformatics.babraham.ac.uk/projects/fastqc/,0.11.9). The HISAT2 (https://daehwankimlab.github.io/hisat2/,version:hisat2-2.2.1) software package was used to align reads from all samples to the *Homo sapiens* reference genome. A database of potential splice junctions was built by HISAT2 and these junctions were confirmed by comparing previously unmapped reads to a database of putative junctions. The reconstruction of transcripts was carried out with software StringTie (http://ccb.jhu.edu/software/stringtie/,version:stringtie-2.1.6). To identify the new transcripts, all of the reconstructed transcripts were aligned to reference genome and were divided into 12 categories by using gffcompare software(http://ccb.jhu.edu/software/stringtie/gffcompare.shtml,version:gffcompare-0.9.8). Transcripts with one of the classcodes “i, j, o, u, x” were defined as novel transcripts, which was then used to predict lncRNA. Of these, transcripts shorter than 200 nt were discarded. Then, the CPC0.9-r2 (http://cpc2.cbi.pku.edu.cn) and CNCI2.0 (https://github.com/www-bioinfo-org/CNCI#install-cnci) with default parameters were utilized to predict novel transcripts with coding potential. All transcripts with CPC score < 0.5 and CNCI score < 0 were retained and considered as novel lncRNAs. The remaining transcripts with class code (I, j, o, u, x) were considered as novel lncRNAs. StringTie (https://ccb.jhu.edu/software/stringtie/, version:stringtie-2.1.6) and gffcompare software (https://ccb.jhu.edu/software/stringtie/gffcompare.shtml, version: gffcompare-0.9.8) were used to generate the final transcriptome. StringTie and ballgown (https://www.bioconductor.org/packages/release/bioc/html/ballgown.html) were used to estimate the expression levels of all transcripts and perform expression abundance for transcripts by calculating FPKM. Differential expression analysis was performed by DESeq2 software between two different groups. The mRNAs/lncRNAs with the parameter of false discovery rate (FDR) below 0.05 and absolute FC ≥ 2 were considered differentially expressed. Differentially expressed coding RNAs were then subjected to enrichment analysis of GO functions and KEGG pathways.

### 2.6. Construction of lncRNA–mRNA Co-Expression Network and Circos Plot

Screening for significantly DElncRNAs and DEmRNAs was conducted from their differential expression profiles to explore the association between mRNA and lncRNA. Pearson correlation coefficients were obtained using the biological website (http://www.bioinformatics.com.cn/). Screen for statistically significant DElncRNAs and DEmRNAs with *p* value < 0.05, *r* ≥ 0.4 or *r*  ≤  −0.4. Finally, the data were imported into Cytoscape software (version 3.5.1) to obtain the lncRNA–mRNA co-expression network for visualization and analysis. A visual display of gene transcription and regulatory data was provided by Circos plots drawn using the RCircos software package. Based on the chromosomal association of lncRNA with mRNA molecules, correlation coefficients greater than 0.95 were considered to indicate a significant correlation between lncRNAs and mRNAs.

### 2.7. ROC Curve Analysis

Potential biomarkers for PMOP were screened for ROC curve analysis based on correlation analysis of DElncRNAs and DEmRNAs. ROC curves were generated using SPSS 22.0 software (IBM, Chicago, IL, USA). We calculated the AUC, sensitivity, specificity, and *p* value of the models to evaluate the diagnostic efficacy of potential lncRNAs for PMOP.

### 2.8. qRT-PCR

About 1 *μ*L of total RNAs from each RNA sample was used to prepare cDNA samples through RT using HiFiScript gDNA Removal RT MasterMix (CWBIO, cat. Cw2020M, China) under conditions of 15 min at 37°C and 5 s at 85°C. Use qPCR to analyze the expression of DElncRNAs with 18S rRNA as an internal control. All the PCR reactions were performed in three replicates, and the relative expression of genes was calculated using 2^−∆∆Ct^, with Ct values normalized by the delta Ct method. PCR thermal conditions were 5 min at 95°C, followed by 40 cycles of 10 s at 95°C (YESEN, cat. 11202ES08, China). The primer sequences for the qRT-PCR assays are listed in Table 1.

### 2.9. Statistical Analysis

SPSS 22.0 software was used for statistical analysis and image preparation. The two-tailed Student's *t*-test was used for continuous variables that satisfy normal distribution and homogeneity of variance. Quantitative data were expressed as mean ± standard deviation (mean ± SD). The relationship between DElncRNA and DEmRNA was verified using Pearson correlation analysis. ROC curve analysis was used for diagnostic analysis. A *p* value < 0.05 was deemed as statistically significant.

## 3. Result

### 3.1. Clinical Characteristics of the Participants

The characteristics of the participants in the study are shown in Table 2. There was no significant difference found between PMOP and n-PMOP controls in terms of participant characteristics such as age, BMI, calcium, or ALP (*p* > 0.05). Besides BMD, the differences in biochemical indices between the PMOP and n-PMOP controls did not reach statistical significance (*p* > 0.05).

### 3.2. Expression Profiling Data of lncRNAs and mRNAs

**Figure 2:**
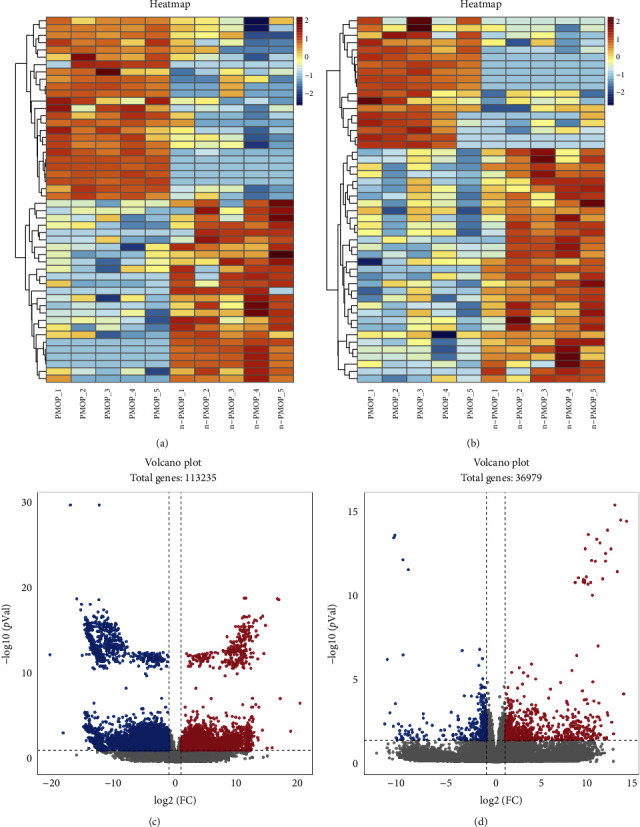
(a) Heatmap for visualization of the top 50 upregulated and downregulated DElncRNAs. (b) Heatmap for visualization of the top 50 upregulated and downregulated DEmRNAs. (c) Volcano plot for visualization of lncRNAs. (d) Volcano plot for visualization of mRNAs.

RNA sequencing technology was used to obtain expression profiling data of lncRNAs and mRNAs in PBMC cells from patients with PMOP and no PMOP. DElncRNAs and DEmRNAs were screened from the date of the expression profiles with |Log_2_FC| > 1 and *p* < 0.05 for subsequent analyses. A total of 1978 DElncRNAs and 1024 DEmRNAs were screened. Compared with the n-PMOP controls, 389 lncRNAs and 704 mRNAs were significantly upregulated and 1589 lncRNAs and 320 mRNAs were significantly downregulated in the PMOP patients. Volcano plot and heatmap were constructed to demonstrate the profiles of the DElncRNAs and DEmRNAs (Figure 2).

DElncRNAs and DEmRNAs were identified using volcano and heat map analysis. Statistical significance was defined as adjusted *p* value < 0.05 and biological significance was defined as FC > 2.0. In volcano plot, red dots refer to upregulated genes, blue dots refer to downregulated genes, and gray dots refer to genes with no significant differences. The smaller the *p* value, the more significant the difference. In heatmap, red color refers to high gene expression, and blue color refers to low expression. The redder the color, the higher the expression, and the bluer the color, the lower the expression. The horizontal coordinate refers to the samples and the vertical coordinate is the tree diagrams of the gene clustering analysis.

### 3.3. Functional Enrichment Analysis of DEmRNAs

**Figure 3:**
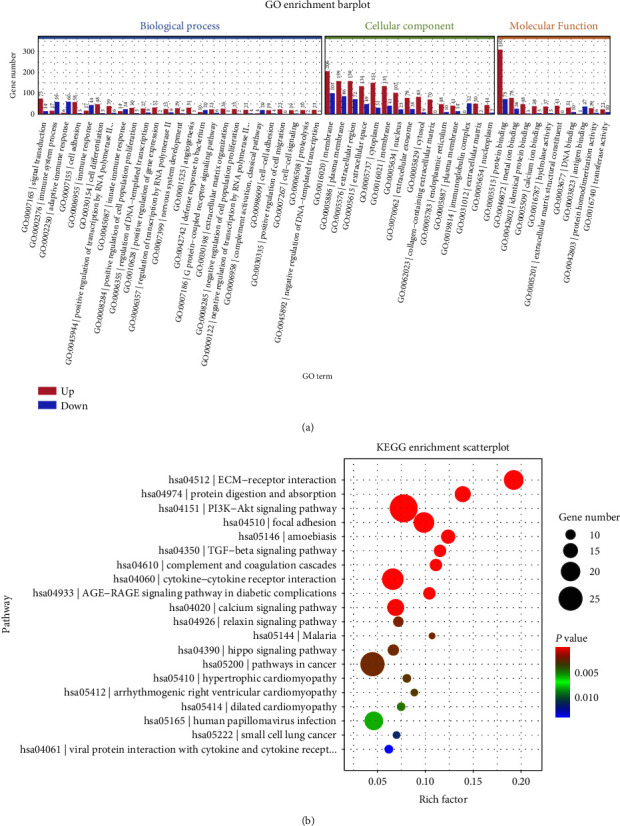
Bioinformatics analysis based on DEmRNAs. (a) GO analysis of DEmRNAs. (b) KEGG analysis of DEmRNAs.

We next performed the functional enrichment analysis on the DEGs. The barplots results are sorted by *p* value, while the scatter plots are ranked based on the counts of enriched genes. GO enrichment analysis indicated that the top 10 biological enriched functions were “protein binding,” “membrane,” “plasma membrane,” “extracellular region,” “extracellular space,” “cytoplasm,” “nucleus,” “metal ion binding,” “signal transduction,” “immune system process,” and “extracellular exosome.” For KEGG enrichment analysis, the key enriched pathways for DElncRNAs were “PI3K-Akt signaling pathway,” “Metabolic pathways,” “Pathways in cancer,” “Focal adhesion,” “Cytokine-cytokine receptor interaction,” “ECM-receptor interaction,” “Human papillomavirus infection,” “Calcium signaling pathway,” and “Neuroactive ligand-receptor interaction” (Figure 3).

### 3.4. Construction of the mRNA–lncRNA Co-Expression Network and Circos Plot

**Figure 4:**
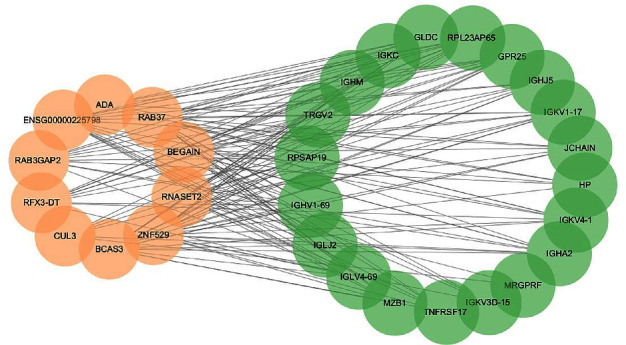
Correlation analysis of DElncRNAs and DEmRNAs. In total, 20 DEmRNAs and 10 DElncRNAs were screened. Green refers to mRNA, and yellow refers to lncRNA. lncRNA RAB37, lncRNA BEGAIN, and lncRNA ZNF529 had the highest number of nodes, 19 in total, followed by 15 nodes of ENSG00000225798 and BCAS3.

**Figure 5:**
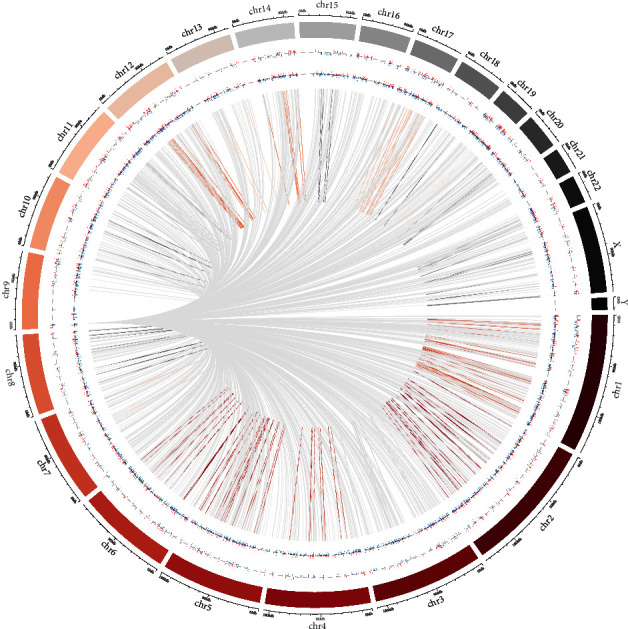
Visualization of transcription and regulation data using the Circos plot.

Based on the expression profiling data completeness and the value of P_adj_, we further minimized the selection. Finally, we screened 10 DElncRNAs that might be important for bone loss (Table 3). Subsequently, we selected 20 DEmRNAs and 10 DElncRNAs to co-construct a correlation network to understand the role and functional mechanism of lncRNAs in PMOP (Figure 4). Among them, lncRNA RAB37, lncRNA BEGAIN, and lncRNA ZNF529 had the highest number of nodes, 19 in total, followed by 15 nodes of ENSG00000225798 and BCAS3. Meanwhile, Circos plots were created to show the role of 10 DElncRNAs and 20 DEmRNAs in PMOP pathology (Figure 5).

Chromosome numbers are located in the outermost layer. The second outermost layer represents all DEmRNAs, and the third layer represents all DElncRNAs. Finally, the inner lines represent the cis or trans lncRNA actions on the target mRNAs.

### 3.5. Validation of the Expression Levels and the Diagnostic Values of DElncRNAs

**Figure 6:**
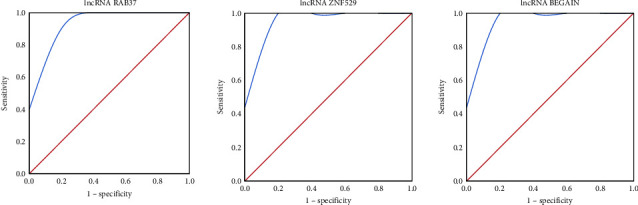
ROC curves to evaluate the diagnostic efficacy of potential lncRNAs.

Based on the bioinformatics analysis after sequencing, 3 DElncRNAs (lncRNA RAB37, lncRNA ZNF529, and lncRNA BEGAIN), which had a strong correlation with DEmRNAs, were selected for the ROC curve analysis. All three key lncRNAs showed acceptable independent diagnostic efficacy. The AUCs of lncRNA RAB37, lncRNA ZNF529, and lncRNA BEGAIN for the diagnosis of PMOP were 0.960 (95% CI: 0.843–1.000), 1.000 (95% CI: 1.000–1.000) and 1.000 (95% CI: 1.000–1.000), respectively. In a small sample setting, lncRNA RAB37, lncRNA BEGAIN, and lncRNA ZNF529 are all highly diagnostic for PMOP (Figure 6).

### 3.6. External Validation of qPCR

**Figure 7:**
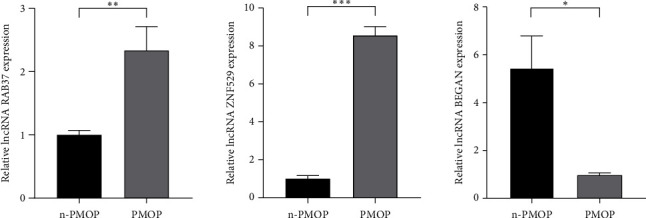
Expression of lncRNA RAB37, lncRNA ZNF529, and lncRNA BEGAIN in normal bone mass and PMOP groups. ⁣^∗^*p* < 0.05; ⁣^∗∗^*p* < 0.01; ⁣^∗∗∗^*p* < 0.001.

Twenty PMOP patients and 20 women with normal bone mass were reincluded for external validation of lncRNAs. DElncRNA expression in PBMC from PMOP patients (*n* = 20) and healthy controls (*n* = 20) was measured by RT-qPCR. lncRNA RAB37, lncRNA ZNF529, and lncRNA BEGAIN were all significantly associated with PMOP development. Of these, lncRNA RAB37 and lncRNA ZNF529 were highly expressed, whereas lncRNA BEGAIN was lowly expressed compared to n-PMOP, consistent with the above sequencing data. The significant difference of lncRNA ZNF529 was superior to that of other lncRNAs. Combined with the above bioinformatics analysis, we concluded that lncRNA ZNF529 is more suitable as a disease diagnostic marker for PMOP (Figure 7).

## 4. Discussion

Women's inherently lower peak bone mass, as well as changes in hormone levels after menopause, can accelerate the body's bone loss [25]. Reduction of estrogen disrupts bone homeostasis, enhancing bone resorption activity and leading to high turnover rate of bone loss [26]. As early as 1994, the World Health Organization considered that women within 5 years of the menopause were worthwhile to be screened for osteoporosis [27]. However, the complexity of osteoporosis itself, the limitations of diagnostic means, and the incomplete public awareness have led to a continued rise in the incidence of osteoporosis in an aging society.

Circulating lncRNA has attracted increasing attention due to the relative simplicity and noninvasiveness of its acquisition and prominence in disease diagnosis [28, 29]. Of the more than 173,000 lncRNAs discovered, only some are well annotated, and there are still a large number of lncRNAs that are worth exploring in depth [30]. In recent years, lncRNAs have shown a strong regulatory role in osteoporosis. lncRNAs can regulate multiple processes of bone metabolism affecting the development of osteoporosis, including osteogenic differentiation of bone marrow mesenchymal stem cells, apoptosis, and alterations in the bone marrow niche such as inflammation [31–33].

The aim of this study was to identify specific lncRNAs associated with PMOP by RNA sequencing and to assess their feasibility as potential biomarkers at the RNA level by combining bioinformatics analysis, diagnostic efficacy assessment, and qPCR.

The deep RNA sequencing we used to explore the lncRNA expression profiles of PMOP patients revealed more dysregulated lncRNAs, especially novel lncRNAs. In this study, a total of 1978 DElncRNAs were screened, with 389 lncRNAs significantly upregulated and 1589 lncRNAs significantly downregulated in PMOP patients compared with n-PMOP controls. GO enrichment analysis indicated that the biologically enriched functions were “protein binding,” “membrane,” “plasma membrane,” etc. In contrast, KEGG analysis revealed that the key enriched pathways for DElncRNAs were “PI3K-Akt signaling pathway,” “Metabolic pathways,” “Pathways in cancer,” “Focal adhesion,” etc. The above may be targets and pathways where DElncRNAs function. Subsequently, we screened the DEmRNAs in the reference transcriptome. Using Pearson correlation coefficients, we established a lncRNA–mRNA co-expression network and identified the number of nodes between each potential DElncRNAs and DEmRNAs. The results showed that lncRNA RAB37, lncRNA ZNF529, and lncRNA BEGAIN had the most nodes with the screened DEmRNAs. Afterward, ROC curves were performed to analyze the diagnostic efficacy of each potential lncRNA for PMOP. In a small sample setting, lncRNA RAB37, lncRNA ZNF529, and lncRNA BEGAIN all showed good diagnostic efficacy. Subsequently, 20 PMOP patients and 20 women with normal bone mass were reincluded for external validation of lncRNAs. The results showed that lncRNA RAB37, lncRNA ZNF529, and lncRNA BEGAIN were all significant in the diagnosis of PMOP, but the significant difference of lncRNA ZNF529 was superior to other lncRNAs.

Zinc finger (ZNF) protein genes are the largest family of transcription factors encoding the human genome [34]. Su et al. [35] performed whole genome sequencing analysis of 4982 patients with osteoporosis and found that ZNF family genes were positively associated with BMD in different skeletal sites and classified them as novel bone-associated genes. Furthermore, ZNFs are well known for their role in the bone marrow microenvironment, which can assist hematopoietic stem cells to efficiently utilize Zn2+ to maintain hematopoietic homeostasis and regulate the activation of the NF-kb pathway, thereby regulating bone reconstruction [36, 37]. Currently, ZNF-related studies primarily focus on bone marrow hematopoiesis and vascular function, while angiogenesis plays an important role in bone metabolism. It may be an essential pathway through which lncRNA ZNF529 affects PMOP. In conclusion, the expression level of lncRNA ZNF529 in PMOP PBMC was significantly higher than that of n-PMOP, and the co-expression network, ROC curve analysis, and q-PCR external validation showed a strong correlation between lncRNA ZNF529 and PMOP, which is expected to be a novel potential biomarker for the clinical diagnosis of PMOP. However, our study has some limitations. The small sample size is susceptible to patient individualization, which may lead to reduced statistical power. Although this study annotated the key target gene functions and signaling pathways associated with DElncRNA, further research and validation have not yet been performed, which is the focus of our subsequent studies. The identification of DElncRNAs may provide new directions to reflect the overall state of bone metabolism and the dynamic process of BMD changes. The lncRNA ZNF529 is highly associated with the development of PMOP and is a potential clinical diagnostic biomarker for PMOP.

## Figures and Tables

**Table 1:** Primers used for quantitative PCR of lncRNAs.

Gene	Primer sequence (5′–3′)	Reverse sequence (5′–3′)
BEGAIN	AGGATTCAGAGCAACTACATGG	ATTGCAGTCCTTCCTATAGAGC
ZNF529	TGGGAAATCCTTTAGAGTGCAT	CCTTGCCACATTCCATACATTT
RAB37	TGTGGATGGCGTGAGAGTGAAG	TGTTGGTGATGTCATACAGCAGAAG
GAPDH	ACACCCACTCCTCCACCTTTG	TCCACCACCCTGTTGCTGTAG

**Table 2:** General clinical data of participants.

Variable	Discovery phase			Validation phase		
	PMOP	n-PMOP	*p* value	PMOP	n-PMOP	*p* value
Number	5	5	—	20	20	—
Age	69.2 ± 6.8	68.2 ± 2.9	0.769	67.8 ± 3.9	62.5 ± 8.34	0.297
BMI	26.5 ± 3.6	23.9 ± 6.9	0.479	22.8 ± 3.44	27.7 ± 4.41	0.132
Age at menopause	52.4 ± 3.1	51.5 ± 4.4	0.527	53.9 ± 3.3	52.7 ± 5.2	0.564
Menopausal duration	23.2 ± 5.8	21.4 ± 6.7	0.428	19.9 ± 4.9	22.8 ± 3.1	0.463
T-score	−2.9 ± 0.5	−1.6 ± 0.5	0.003	−2.8 ± 0.3	0.47 ± 1.2	0.002
25(OH)D	45.0 ± 9.3	55.9 ± 16.7	0.235	35.1 ± 17.2	50.8 ± 16.2	0.232
ALP	78.2 ± 27.2	73.4 ± 13.7	0.733	54.5 ± 10.9	82.8 ± 26.1	0.351
Calcium	2.3 ± 0.1	2.2 ± 0.1	0.374	2.5 ± 0.1	2.4 ± 0.1	0.093

*Note:* Serum samples were obtained from whole blood and tested in the same laboratory for bone metabolism indices such as calcium, ALP, and 25(OH)D. Data are represented as mean ± standard deviation. All *p* values were calculated with the *t*-test. *p* value < 0.05 was considered to indicate a statistically significant difference. BMI, body mass index; 25(OH)D, 25-hydroxy vitamin D; ALP, alkaline phosphatase.

**Table 3:** Top 10 of DElncRNAs in patients with PMOP compared to n-PMOP.

Gene name	Gene ID	Chr	Log_2_FC	*p* value	*P*_adj_
RAB37	MSTRG.43194	17	3.00	2.10 × 10^−6^	1.06 × 10^−4^
BEGAIN	MSTRG.32489	14	−2.54	1.44 × 10^−5^	7.14 × 10^−4^
RNASET2	MSTRG.84713	6	1.93	3.36 × 10^−5^	1.64 × 10^−3^
ZNF529	MSTRG.47832	19	2.34	4.07 × 10^−5^	1.98 × 10^−3^
BCAS3	MSTRG.42583	17	−2.44	4.68 × 10^−5^	2.26 × 10^−3^
CUL3	MSTRG.56862	2	4.59	4.69 × 10^−5^	2.27 × 10^−3^
RFX3-DT	MSTRG.94341	9	−2.17	8.01 × 10^−5^	3.80 × 10^−3^
RAB3GAP2	MSTRG.11889	1	2.20	1.87 × 10^−4^	8.43 × 10^−3^
ENSG00000225798	MSTRG.34443	15	2.96	2.23 × 10^−4^	9.92 × 10^−3^
ADA	MSTRG.59116	20	−1.14	2.51 × 10^−4^	1.10 × 10^−2^

*Note:* Top 10 lncRNAs were selected based on the rank of absolute value of *P*_adj_.

## Data Availability

The data that support the findings of this study are available on request from the corresponding author. The data are not publicly available due to privacy or ethical restrictions.

## References

[1] Walker M. D., Shane E. (2023). Postmenopausal Osteoporosis. *New England Journal of Medicine*.

[2] Arceo-Mendoza R. M., Camacho P. M. (2021). Postmenopausal Osteoporosis: Latest Guidelines. *Endocrinology and Metabolism Clinics of North America*.

[3] Wright N. C., Looker A. C., Saag K. G. (2014). The Recent Prevalence of Osteoporosis and Low Bone Mass in the United States Based on Bone Mineral Density at the Femoral Neck or Lumbar Spine. *Journal of Bone and Mineral Research*.

[4] Mo X., Zhao S., Wen Z. (2021). High Prevalence of Osteoporosis in Patients Undergoing Spine Surgery in China. *BMC Geriatrics*.

[5] Eastell R., Szulc P. (2017). Use of Bone Turnover Markers in Postmenopausal Osteoporosis. *Lancet Diabetes & Endocrinology*.

[6] Fischer V., Haffner-Luntzer M. (2022). Interaction Between Bone and Immune Cells: Implications for Postmenopausal Osteoporosis. *Seminars in Cell & Developmental Biology*.

[7] Xu Q., Li D., Chen J. (2022). Crosstalk Between the Gut Microbiota and Postmenopausal Osteoporosis: Mechanisms and Applications. *International Immunopharmacology*.

[8] Cheng C. H., Chen L. R., Chen K. H. (2022). Osteoporosis Due to Hormone Imbalance: An Overview of the Effects of Estrogen Deficiency and Glucocorticoid Overuse on Bone Turnover. *International Journal of Molecular Sciences*.

[9] Lipiński P., Stępień K. M., Ciara E., Tylki-Szymańska A., Jezela-Stanek A. (2021). Skeletal and Bone Mineral Density Features, Genetic Profile in Congenital Disorders of Glycosylation: Review. *Diagnostics*.

[10] Dougherty J., Jiang X., Schnatz P. F. (2016). Risk Assessment Tools for Osteoporosis-Related Fractures. *Connecticut Medicine*.

[11] Camacho P. M., Petak S. M., Binkley N. (2020). American Association Of Clinical Endocrinologists/American College of Endocrinology Clinical Practice Guidelines for the Diagnosis aAnd Treatment of Postmenopausal Osteoporosis-2020 Update. *Endocrine Practice*.

[12] Goodwin S., McPherson J. D., McCombie W. R. (2016). Coming of Age: Ten Years of Next-Generation Sequencing Technologies. *Nature Reviews Genetics*.

[13] Herman A. B., Tsitsipatis D., Gorospe M. (2022). Integrated lncRNA Function Upon Genomic and Epigenomic Regulation. *Molecular Cell*.

[14] Bridges M. C., Daulagala A. C., Kourtidis A. (2021). LNCcation: lncRNA Localization and Function. *Journal of Cell Biology*.

[15] Graf J., Kretz M. (2020). From Structure to Function: Route to Understanding lncRNA Mechanism. *BioEssays*.

[16] Yan Y., Liu J., Xu Z., Ye M., Li J. (2021). lncRNA PCAT14 Is a Diagnostic Marker for Prostate Cancer and is Associated With Immune Cell Infiltration. *Disease Markers*.

[17] Kuang S., Wang J., Wei Z., Zhai F., Liang S. (2023). The Regulatory Function of lncRNA and Constructed Network in Epilepsy. *Neurological Sciences*.

[18] Okuyan H. M., Begen M. A. (2022). LncRNAs in Osteoarthritis. *Clinica Chimica Acta*.

[19] Wu C., Wang C., Xiao B. (2024). Integration Analysis of lncRNA and mRNA Expression Data Identifies DOCK4 as a Potential Biomarker for Elderly Osteoporosis. *BMC Medical Genomics*.

[20] Yu C., Chen B., Su H., Yang Y. (2024). Long Non-Coding RNA MIAT Serves as a Biomarker of Fragility Fracture and Promotes Fracture Healing. *Journal of Orthopaedic Surgery and Research*.

[21] Yang Y., Yujiao W., Fang W. (2020). The Roles of miRNA, lncRNA and circRNA in the Development of Osteoporosis. *Biological Research*.

[22] Zhu X. X., Yan Y. W., Chen D. (2016). Long Non-Coding RNA HoxA-AS3 Interacts With EZH2 to Regulate Lineage Commitment of Mesenchymal Stem Cells. *Oncotarget*.

[23] Zhang Z., Meng Y., Lin T. (2024). Dancr-BRG1 Regulates Nfatc1 Transcription and Pgc1*β*-Dependent Metabolic Shifts in Osteoclastogenesis. *Proceedings of the National Academy of Sciences*.

[24] Wumiti T., Wang L., Xu B. (2024). lncTIMP3 Promotes Osteogenic Differentiation of Bone Marrow Mesenchymal Stem Cells via miR-214/Smad4 Axis to Relieve Postmenopausal Osteoporosis. *Molecular Biology Reports*.

[25] Weaver C. M., Gordon C. M., Janz K. F. (2016). The National Osteoporosis Foundation’s Position Statement on Peak Bone Mass Development and Lifestyle Factors: a Systematic Review and Implementation Recommendations. *Osteoporosis International*.

[26] Wang L. T., Chen L. R., Chen K. H. (2023). Hormone-Related and Drug-Induced Osteoporosis: A Cellular and Molecular Overview. *International Journal of Molecular Sciences*.

[27] Kanis J. A., Kanis J. A. (1994). Assessment of Fracture Risk and its Application to Screening for Postmenopausal Osteoporosis: Synopsis of a WHO Report. *Osteoporosis International*.

[28] Kishikawa T., Otsuka M., Ohno M., Yoshikawa T., Takata A., Koike K. (2015). Circulating RNAs as New Biomarkers for Detecting Pancreatic Cancer. *World Journal of Gastroenterology*.

[29] Xiao Y., Xiao T., Ou W. (2020). LncRNA SNHG16 as a Potential Biomarker and Therapeutic Target in Human Cancers. *Biomarker Research*.

[30] Yip C. W., Sivaraman D. M., Prabhu A. V., Shin J. W. (2021). Functional Annotation of lncRNA in High-Throughput Screening. *Essays in Biochemistry*.

[31] Yang Y., Miao L., Chang S. (2022). Exosome-Derived LncRNA TCONS_00072128 Mediated Osteogenic Differentiation and Inflammation by Caspase 8 Regulation. *Frontiers in Genetics*.

[32] Huang X., Jie S., Li W., Liu C. (2023). GATA4-Activated lncRNA MALAT1 Promotes Osteogenic Differentiation Through Inhibiting NEDD4-Mediated RUNX1 Degradation. *Cell death discovery*.

[33] Liu H., Wang Y. W., Chen W. D., Dong H. H., Xu Y. J. (2021). Iron Accumulation Regulates Osteoblast Apoptosis Through lncRNA XIST/miR-758-3p/caspase 3 Axis Leading to Osteoporosis. *IUBMB Life*.

[34] Sun X., Zheng D., Guo W. (2022). Comprehensive Analysis of a Zinc Finger Protein Gene-Based Signature With Regard to Prognosis and Tumor Immune Microenvironment in Osteosarcoma. *Frontiers in Genetics*.

[35] Su K. J., Qiu C., Greenbaum J. (2024). Genomic Structural Variations Link Multiple Genes to Bone Mineral Density in a Multi-Ethnic Cohort Study: Louisiana Osteoporosis Study. *Journal of Bone and Mineral Research*.

[36] da Silva Lima F., da Silva Gonçalves C. E., Fock R. A. (2023). A Review of the Role of Zinc Finger Proteins on Hematopoiesis. *Journal of Trace Elements in Medicine & Biology*.

[37] Martens A., Hertens P., Priem D. (2022). A20 Controls RANK-Dependent Osteoclast Formation and Bone Physiology. *EMBO Reports*.

